# A fluorescence lifetime separation approach for FLIM live‐cell imaging

**DOI:** 10.1111/jmi.70036

**Published:** 2025-09-30

**Authors:** Cornelia Wetzker, Marcelo Leomil Zoccoler, Svetlana Iarovenko, Chukwuebuka William Okafornta, Anja Nobst, Hella Hartmann, Thomas Müller‐Reichert, Robert Haase, Gunar Fabig

**Affiliations:** ^1^ B CUBE – Center for Molecular Bioengineering Technische Universität Dresden Dresden Germany; ^2^ Light Microscopy Facility Technology Platform Center for Molecular and Cellular Bioengineering Technische Universität Dresden Dresden Germany; ^3^ DFG Cluster of Excellence ‘Physics of Life’ (PoL) Technische Universität Dresden Dresden Germany; ^4^ Institute of Molecular Biotechnology of the Austrian Academy of Sciences (IMBA) Vienna BioCenter (VBC) Vienna Austria; ^5^ Experimental Center Faculty of Medicine Carl Gustav Carus Technische Universität Dresden Dresden Germany; ^6^ Core Facility Cellular Imaging (CFCI) Faculty of Medicine Carl Gustav Carus Technische Universität Dresden Dresden Germany; ^7^ Center for Scalable Data Analytics and Artificial Intelligence (ScaDS.AI) Dresden/Leipzig Germany; ^8^ Faculty of Mathematics and Computer Science Leipzig University Leipzig Germany

**Keywords:** FLIM, live‐cell imaging, napari, phasor, OMERO

## Abstract

Fluorescence lifetime imaging microscopy (FLIM) translates the duration of excited states of fluorophores into lifetime information as an additional source of contrast in images of biological samples. This offers the possibility to separate fluorophores particularly beneficial in case of similar excitation spectra. Here, we demonstrate the distinction of fluorescent molecules based on FLIM phasor analysis, called lifetime separation, in live‐cell imaging using open‐source software for analysis. We showcase two applications using *Caenorhabditis elegans* as a model system. First, we separated the highly spectrally overlapping fluorophores mCherry and mKate2 to distinctively track tagged proteins in six‐dimensional datasets to investigate cell division in the developing early embryo. Second, we separated fluorescence of tagged proteins of interest from masking natural autofluorescence in adult hermaphrodites. For FLIM data handling and workflow implementation, we developed the open‐source plugin napari‐flim‐phasor‐plotter to implement conversion, visualisation, analysis and reuse of FLIM data of different formats. Our work thus advances technical applications and bioimage data management and analysis in FLIM microscopy for life science research.

## LAY DESCRIPTION

Fluorescence lifetime imaging microscopy abbreviated as FLIM is a specialised application of microscopy in which the duration from the uptake or absorption of a photon to the release or emission by a fluorescent molecule is of central importance. This excited higher energetic state only lasts pico‐ to nanoseconds for many fluorescent proteins and thus requires the usage of lasers pulsed at Megahertz repetition rates, fast detectors capable to detect single molecules as well as fast electronics. We applied such a setup called FLIM microscope to distinguish either two red fluorophores that have nearly identical excitation and emission spectra in living biological samples. If coexistent in an investigated sample, both fluorophores would always be excited for fluorescence simultaneously and could not be told apart.

We used FLIM microscopy to image cellular proteins with fluorescent tags being either mCherry or mKate2 in embryos of the nematode *Caenorhabditis elegans* during its first cell divisions. To achieve this, we developed a python‐based open‐source software plugin called napari‐flim‐phasor‐plotter (NFPP, https://napari-flim-phasor-plotter.readthedocs.io/en/stable/intro.html) that reads FLIM images of various formats and transforms the lifetime information of each position in our multidimensional images into a data point of a two‐dimensional phasor plot. This phasor analysis clusters points of our image datasets with similar fluorescent content in the plot and thus allows selection of image positions of similar biological origin. This approach allowed us to distinguish and further analyse the movements of proteins of either mCherry or mKate2 in our six‐dimensional datasets separately. The dimensions include three spatial dimensions in *x*, *y* and *z* with sequential acquisition in two spectral ranges including the dimension of lifetime information and followed as time‐series for several minutes. In a second experiment, we separated proteins tagged with green fluorescent protein (GFP) from natural autofluorescence of the gut in the green range in adult hermaphrodite worms based on their lifetime information.

We visualise and share openly the datasets of our experiments annotated with experimental and technical metadata in the BioImage Archive (https://www.ebi.ac.uk/biostudies/bioimages/studies/S‐BIAD1967) and using the bioimage management platform OMERO (https://www.openmicroscopy.org/omero/) for all to inspect and reuse (https://omero.med.tu‐dresden.de/webclient/?show=project‐1401).

Our work thus simplifies the usage of FLIM microscopy and the analysis of its data particularly with open‐source software and enhances sharing and reuse of microscopy data in biological and biomedical research.

## 1 INTRODUCTION

Fluorescence microscopy is broadly applied for the investigation of molecular processes in living samples. Largely based on spectrally distinct endogenous or extrinsic fluorophores, it enables the relatively precise localisation of subcellular structures in space and time. Due to the spectral overlap of excitation and emission spectra of fluorophores and the limitation of available laser lines and tuneable laser ranges, the number of simultaneously investigated cellular features is technically restricted. However, multiplexed simultaneous imaging of multiple cellular features is crucial for an understanding of the complex interplay of biomolecules in cells and tissues.

These limitations can be overcome partially by the application of specialised imaging techniques such as spectral unmixing. Based on dispersion of emitted light, the latter can be used in cases of minimal differences in either excitation or emission spectra or both.^1^ However, the technique requires adequate single fluorophore controls. The assessment of fluorescence lifetime in bioimages, termed fluorescence lifetime imaging microscopy (FLIM), is an alternative approach. In the past decades, FLIM has been mostly applied to investigate physical environments of fluorophores, aspects of cellular metabolism and also protein‐protein interactions across various species and biological samples.^2^, ^3^, ^4^ It gains further importance for the separation of different fluorophore signals in fixed samples which we term lifetime separation.^5^, ^6^, ^7^

The fluorescence lifetime being the duration of the excited state upon photon absorption by the fluorochrome is a characteristic attribute of each fluorophore in its microenvironment.^2^ This source of contrast can be used to classify pixels or objects in bioimaging. A disadvantage of FLIM is the requirement for higher photon counts for an accurate lifetime decay curve representation and analysis which may require extended image acquisition partially at the cost of temporal and spatial resolution.^8^, ^9^ Yet, advancements in laser technology, steady improvement in the sensitivity of detectors and an increase in the number of pulsed lasers in light microscopy facilities have contributed to a higher popularity of this technique and a demand for improved data handling and analysis workflows suitable for specific applications.

Besides the broadly applied exponential fitting of lifetime decays, phasor analysis represents an alternative mode of analysis.^10^, ^11^, ^12^ It generates graphical representations of lifetime characteristics per pixel of an image by Fourier transformation of decay data into two‐dimensional (2D) plots without the investigator's assumption of the number of lifetime components of the sample in the experimental scenario. Data points from pixels of similar biological content form clusters in phasor plot visualisations and allow classification of corresponding pixels as one entity for further analysis. In addition, phasor analysis allows the estimation of absolute lifetimes or relative changes between clusters on calibrated setups as well as relative contributions to clusters. Furthermore, phasor analysis is convenient to assess the complexity of distinct fluorophores and fluorochrome states within a given sample.^5^, ^6^, ^13^, ^14^, ^15^, ^16^ Those diverse workflows make FLIM combined with phasor analysis a powerful imaging technology to explore both known and unknown biomolecules in more depth.

In this work, we demonstrate the use of lifetime separation to distinguish signals of fluorophores of nearly identical emission spectra involving conversion and processing of multi‐dimensional FLIM datasets for imaging of dynamic processes. We investigated both early embryos and adult hermaphrodites of the nematode *Caenorhabditis elegans* to showcase two applications of multi‐channel phasor FLIM.

To date, the availability of and access to workflows for handling, analysis and sharing of FLIM image data is limited and partially restricted by proprietary file formats.^17^ Three examples mentioned along this manuscript are: LIF – Leica Image File from the company Leica Microsystems which might contain FastFLIM arrays and pre‐computed phasor coordinates; PTU – a FLIM format created by the company PicoQuant GmbH for time‐correlated single photon counting (TCSPC) data and metadata; and SDT – a FLIM file format created by the company Becker & Hickl for storage of TCSPC data and metadata. Bio‐Formats, a software tool for reading and writing image data, is capable of reading many files like LIF, but more specific files containing raw FLIM data can only be read, to some extent, from code by some libraries.^17^ This limits extensive exploration, critical evaluation and reuse of valuable scientific content for the life science community.

In the past years, there has been continuous effort by the scientific community to produce software capable of analysing FLIM data. FLIMfit^18^ and FLIMJ^19^ offer FLIM analysis via the fitting approach, and recently there has been a number of tools that offer the phasor approach, like PAM,^20^ FLUTE,^21^ PhasorIdentifier,^22^ napari‐live‐flim,^23^ the library phasorpy,^24^ napari‐phasors,^25^ GSLab^26^ and FLIMPA.^27^ Still, none of the abovementioned options supports the processing of high‐dimensional bio‐image datasets. We thus developed an open‐source napari plugin, termed napari‐flim‐phasor‐plotter (NFPP, https://napari-flim-phasor-plotter.readthedocs.io/en/stable/intro.html) to visualise and phasor analyse large high‐dimensional FLIM datasets of various formats.^28^, ^29^ Based on the resulting specific lifetime information clusters of phasor plots, image datasets were processed and analysed with NFPP integrated with other napari plugins and standard open‐source image processing libraries for post‐processing. The plugin further includes the conversion of FLIM data to the chunked file format ZARR for handling N‐dimensional data arrays.^30^ The ZARR format allows lazy loading for faster visualisation and processing using cloud‐based tools. Moreover, we present a practicable workflow to visualise, share and publish 6D FLIM datasets in the bioimage management platform Open Microscopy Environment (OME) Remote Objects (OMERO).^31^, ^32^

## 2 MATERIALS AND METHODS

### 2.1 C. *elegans* maintenance

Animals of different *C. elegans* strains (Table 1) were grown on nematode growth medium (NGM) plates at 20°C with *E. coli* (OP50) as food source according to established protocols.^33^

**TABLE 1:** Transgenic and wild‐type *C. elegans* lines.

Strain name	Genotype	Fluorescently labelled cellular structures	Origin
N2	Wild‐type	none	CGC, Minnesota^33^
LP307	cpIs54 [mex‐5p::mKate2::PLC(delta)‐PH(A735T)::tbb‐2 3'UTR + unc‐119 (+)] II; unc‐119(ed3) III	membrane‐mKate2	CGC, Minnesota^34^
TMR17	ddIs6 [tbg‐1::pP + unc‐119(+)]; ltIs37 [pAA64; pie‐1::mCherry::HIS‐58 + unc‐119(+)] IV; unc‐119(ed3) III	chromatin‐mCherry, centrosome‐GFP	Müller‐Reichert Lab, Dresden^35^
TMR31	dIs6 [tbg‐1::GFP + unc‐119(+)]; ltIs37[pAA64; pie‐1::mCherry::HIS‐58 + unc‐119(+)] IV; cpIs54 [mex‐5p::mKate2::PLC(delta)‐PH(A735T)::tbb‐2 3'UTR + unc‐119 (+)] II; unc‐119(ed3) III	chromatin‐mCherry, membrane‐mKate2, centrosome‐GFP	Müller‐Reichert Lab, Dresden (this study)
AZ212	ruIs32 [pAZ132: pie‐1/GFP/histoneH2B] III; unc‐119(ed3) III	chromatin‐GFP, natural gut autofluorescence	Hyman Lab, Dresden^36^
JJ1473	zuIs45 [nmy‐2::NMY‐2::GFP + unc‐119(+)] V; unc‐119(ed3) III	gonad‐GFP, natural gut autofluorescence	Hyman Lab, Dresden^37^

For imaging of *C. elegans* embryos, lines single‐ or double‐positive for fluorescently tagged proteins, namely an mCherry‐tagged chromosome marker (chromatin‐mCherry, HIS‐58), an mKate2‐tagged membrane marker (membrane‐mKate2, phospholipase C delta) and a GFP‐tagged centrosome marker (centrosome‐GFP, γ‐tubulin chain 1), and a wild‐type negative line (N2) were used.^34^, ^38^, ^39^ For imaging of hermaphrodites, lines expressing a GFP‐tagged chromosome marker (chromosome‐GFP, histone H2B) or a GFP‐tagged gonadal marker (gonad‐GFP, NMY‐2) were used.

### 2.2 Sample preparation for imaging

For imaging of early *C. elegans* embryos, adult hermaphrodites were dissected in M9 buffer containing 25 mM levamisole hydrochloride (MP Biomedicals, Solon, USA). Isolated embryos were mounted on a 4% (w/v) agarose pad between a slide and a coverslip.^40^, ^41^ For visualisation of whole animals, we immobilised the worms in between an agar pad and a coverslip using M9 buffer containing 25 mM levamisole hydrochloride.^42^, ^43^

### 2.3 FLIM microscopy

FLIM was performed on the basis of TCSPC using an upright confocal laser scanning microscope (STELLARIS 8, Leica Microsystems, Germany) with a pulsed supercontinuum laser (NKT Photonics, Germany) for single photon excitation at 40 MHz frequency using a water immersion objective (40x/1.1, HC PL APO CS2, Leica Microsystems, Germany).^44^ We used the acquisition software Leica Application Suite X (LAS X) (version 4.5.0.25531) including the FLIM/FCS license (version 4.5.0). GFP was excited at 480 nm (approximately 16 µW in the sample position with absence of the objective) and emitted in the range of 500 to 560 nm. mCherry and mKate2 were excited either alone or simultaneously at 590 nm (approximately 84 µW in the sample position with absence of the objective) and emitted in the range of 610– 800 nm. The pinhole was set to one Airy unit at 600 nm reference. Both spectral ranges were imaged simultaneously. Emitted light was detected with hybrid HyD X detectors (Leica Microsystems, Germany).

The field of view and zoom factor of 4 were chosen to image whole embryos as *z* stacks of FLIM images of 256 × 256 pixels. The image stacks were recorded with 600 Hz speed of bidirectional scans and optical zoom of 4. This yielded a pixel dwell time of 2.4 µs, a frame time of 0.66 s and voxel sizes of 0.27 µm in x and y and 0.5 µm in the z dimension. To collect sufficient photons for FLIM imaging, three frames were summed per image yielding a total scan time of approximately 40 s or shorter per image stack. Acquisition was performed continuously from two‐cell stage prior to the first division until condensed chromatin in the second division appeared (differing from 18 to 30 min between embryos).

For whole worms we recorded several time points but analysed only a single. Two channels *z* stacks of FLIM images were acquired with the same setup as embryos. Differences in central settings included optical zoom of 1, image sizes of 512 × 128 pixels resulting in voxel sizes of 0.54 µm × 0.54 µm × 0.5 µm in x, y, and z . Pixel dwell times were 1.2 µs and frame times of 0.58 s with two added frames leading to 99.2 s (AZ212) and 116.5 s (JJ1473) per stack per time point. The 6D FLIM image datasets were then saved in LIF format in a containerised data structure. We then exported 6D embryo datasets and 5D single time point adult datasets as individual 4D files in PTU format (dimensions being x, y, channels and photon counts in the FLIM dimension which we refer to as microtime) for each z plane and time point as sole export mode for downstream data processing and sharing.

### 2.4 Scans for excitation spectra of fluorophores

Spectral excitation scan images of one biological replicate per genotype were acquired on the same microscope with same setup as FLIM images in the range of 440 to 790 nm with 67 steps of 5 nm step size using the white‐light laser (wavelength‐dependent power between 13 and 51 µW in non‐objective control) at 40 MHz pulse rate. Emitted photons were detected in a 20 nm spectral window of 10 nm distance of excitation wavelength. Datasets were saved along FLIM images in the corresponding LIF files prior to export. Photon counts were summed and normalised across whole images for each series and plotted for comparison of excitation spectra of fluorophores.

### 2.5 FLIM phasor analysis in napari

The NFPP plugin was designed to load raw FLIM data as a napari image layer, generate a phasor plot in a docked plotter widget and interactively segment regions in the image by selecting clusters in the phasor plot. Profiting from previous experience at developing napari‐clusters‐plotter,^45^ a plugin to perform interactive unsupervised machine learning by selecting features in a plotter and mapping them back to labelled objects, we repurposed its plotter to display G and S coordinates stored in a napari labels layer that labels individual pixels instead of objects. This integration had the benefit of readily bringing clustering algorithms, originally from scikit‐learn^46^ ported to napari‐clusters‐plotter, to the phasor plot.

Phasor calculation was implemented using the fast Fourier transform over the photon count dimension. It uses functions to speed up phasor calculation for in‐memory data, but also allows lazy computation in case of data larger than memory. For loading raw FLIM data, the plugin uses specific python libraries to read a couple individual proprietary FLIM file formats, like PTU and SDT.^47^, ^48^ It can build 3D and higher dimensional stacks from individual files containing planes, and convert files or folders to OME‐TIFF for later OMERO upload or to ZARR for lazy loading of large image data.

### 2.6 Conversion and storage of FLIM image formats

We used NFPP to re‐build each 6D dataset as a single stack from multiple individual 4D PTU files and convert them to single ZARR (https://zarr.dev/) arrays. Each file contained the following dimensions: channel, photon counts per time bin (microtime), time, z, y and x. This conversion to ZARR divides N‐dimensional datasets into so‐called chunks. This allowed lazy loading, an optimised loading into memory of the subset of data needed at a time for increased speed and working with larger‐than‐memory data sizes, into napari.

The complete dataset of raw and processed bioimages is further archived in the public BioImage Archive (https://www.ebi.ac.uk/biostudies/bioimages/studies/S‐BIAD1967).^49^, ^50^ We further deposited the datasets in a public user space of an instance of the bioimage management software OMERO for visual inspection (https://omero.med.tu‐dresden.de/webclient/?show=project‐1401).^51^

Currently, OMERO upload and visualisation are supported for datasets up to 5 dimensions (5D). Hence, visualisation of 6D datasets using open‐source tools requires several steps of reformatting, including reduction to 5D for sharing via OMERO. We transformed 6D ZARR to 5D OME‐TIFF datasets by conversion including separation of individual time points. Here, the time dimension of files stores the lifetime decay information. This allowed OMERO upload and thus visual exploration of the lifetime dimension with a slider via the OMERO web interface.

Finally, we also built an extra single OME‐TIFF file per dataset with the microtime dimension reduced to summed intensity information and time points of the actual time series in the time dimension. Hence, these 5D intensity datasets lacked the fluorescence lifetime information but allowed continuous navigation throughout the whole time‐lapse using a slider with the option of adding segmentation results as an extra channel in OMERO (see example dataset, accessed via https://omero.med.tu‐dresden.de/iviewer/?images=37177&dataset=2037). Bioimage datasets were annotated according to REMBI metadata recommendations.^52^

### 2.7 Lifetime separation based on FLIM phasors

We distinguished the fluorescence signal of the chromatin label mCherry based on its lifetime signature distinct from that of mKate2 using NFPP. We defined a region in the phasor plot that corresponded exclusively to mCherry labelled voxels. This region was selected manually based on the phasor patterns of the single control strains TMR17 and LP307, which contained only mCherry‐labelled histone H2B and mKate2‐labelled membrane marker, respectively. We defined the phasor lifetime signature of mCherry‐tagged proteins by selection of an elliptic field around the single cluster that was broad enough to always encompass centres of clusters over all time‐points but not too large to decrease noise inclusion. This fixed elliptic region was then applied to phasor plots of FLIM datasets from *C. elegans* strains with different tagged proteins in the red spectrum (double positive for mCherry and mKate2, and single positive for mKate2).

This workflow is available in the GitHub repository (https://gitlab.mn.tu‐dresden.de/bia‐pol/lifetime‐unmixing/‐/blob/main/notebooks/phasor_plot_analysis_batch_processing.py?ref_type=heads). Briefly, each dataset converted to the ZARR format, available as an option for large datasets in NFPP, was loaded into napari. Here, phasor plots were generated with a fixed set of parameters: first harmonic, binning applied slice‐by‐slice once with a 3 × 3 kernel, intensity threshold 15 and median filter applied with 3 iterations. The fixed, manually adjusted ellipse selection was applied to generate a new layer of segmentation mask resulting from the selection. To reduce noise, morphological opening, fill holes and minimal object size filtering methods were applied on the masks.^53^, ^54^, ^55^ Finally, instance segmentation retrieved individual objects representing the chromatin, followed by feature extraction to get each object's centroid position and volume for downstream analysis of chromatin dynamics.

This segmentation workflow generated the following output: a new channel complementary to the intensity image over the whole time‐lapse as OME‐TIFF files, phasor and pixel coordinates tables for each time‐point as CSV files, phasor plot screenshots before and after selection for each time‐point as PNG files, objects centroid and volume tables over the whole time lapse as CSV files, and parameters values used as TXT files. These results are available via OMERO. The position of the centroids of the segmented chromosomes was used to calculate the three‐dimensional (3D) Euclidean distance among them in each frame. Then the distances were plotted over time and an exponential function was fitted to the measurements using Python to estimate the initial speed of chromosome segregation.^56^

For the analysis of the pole‐to‐pole distance, the centrosomes were segmented using the GFP fluorescence channel by adaptive local thresholding.^57^ Then the distance was calculated analogously as described above. A polynomial function of first degree was fitted to the pole‐to‐pole distance from anaphase onset (0 s) to 120 s after the initiation of anaphase as this period resembled the linear part of the sigmoid curve. From this, the speed of pole‐to‐pole separation could be estimated.

### 2.8 Analysis of adult worms

We analysed worm lines with GFP‐tagged proteins, namely histone H2B (strain AZ212) and non‐muscle myosin‐2 (NMY‐2, strain JJ1473). The histone H2B‐tagged strain AZ212 is used to follow chromatin dynamics in both gonadal and somatic cells in *C. elegans*. Non‐muscle myosin‐2 is expressed in the worm gonad and is important in cell division.^58^ Present in the gonad of hermaphrodites, it is involved in maintaining the gonads’ structure and the oocyte formation.^59^

A common problem when imaging living animals such as *C. elegans* is autofluorescence, mostly associated with the worm's intestine.^60^, ^61^ In these examples, the lifetime of pixels representing autofluorescence and GFP are quite different, which implies clearly distinct clusters in the phasor plots.

We generated phasor plots of FLIM datasets (third harmonic, no binning, intensity threshold 15 and median filter applied with 3 iterations) and automatically segmented data points of the phasor plot into two distinct clusters using the k‐means clustering algorithm from the scikit‐learn python library through the napari‐clusters‐plotter plugin.^45^, ^46^ We saved the voxels of each cluster as a separate pseudo‐channel and uploaded these results to OMERO. For identification of cell nuclei we used two segmentation workflows: (a) we applied the Voronoi‐Otsu labelling algorithm from pyclesperanto (https://zenodo.org/records/10432619), counted the number of segmented objects, and stored their centroid positions and volume in a table; (b) we used fill holes and morphological operations (area closing, isotropic closing and isotropic opening) on the pseudo‐channel representing the mCherry histone H2B cluster.^62^ Then, we applied again a Voronoi‐Otsu labelling, counted the number of objects, and stored their centroid positions and volumes as well. Both workflows can be visualised and reproduced by running the following notebook in the code repository: https://github.com/zoccoler/Lifetime‐Separation/blob/v0.0.3/code/phasor_plot_AZ212.ipynb. We uploaded the segmentation results as new channels to OMERO along with the number of objects to compare the improvement of accuracy after lifetime separation.

### 2.9 Data presentation

**FIGURE 1:**
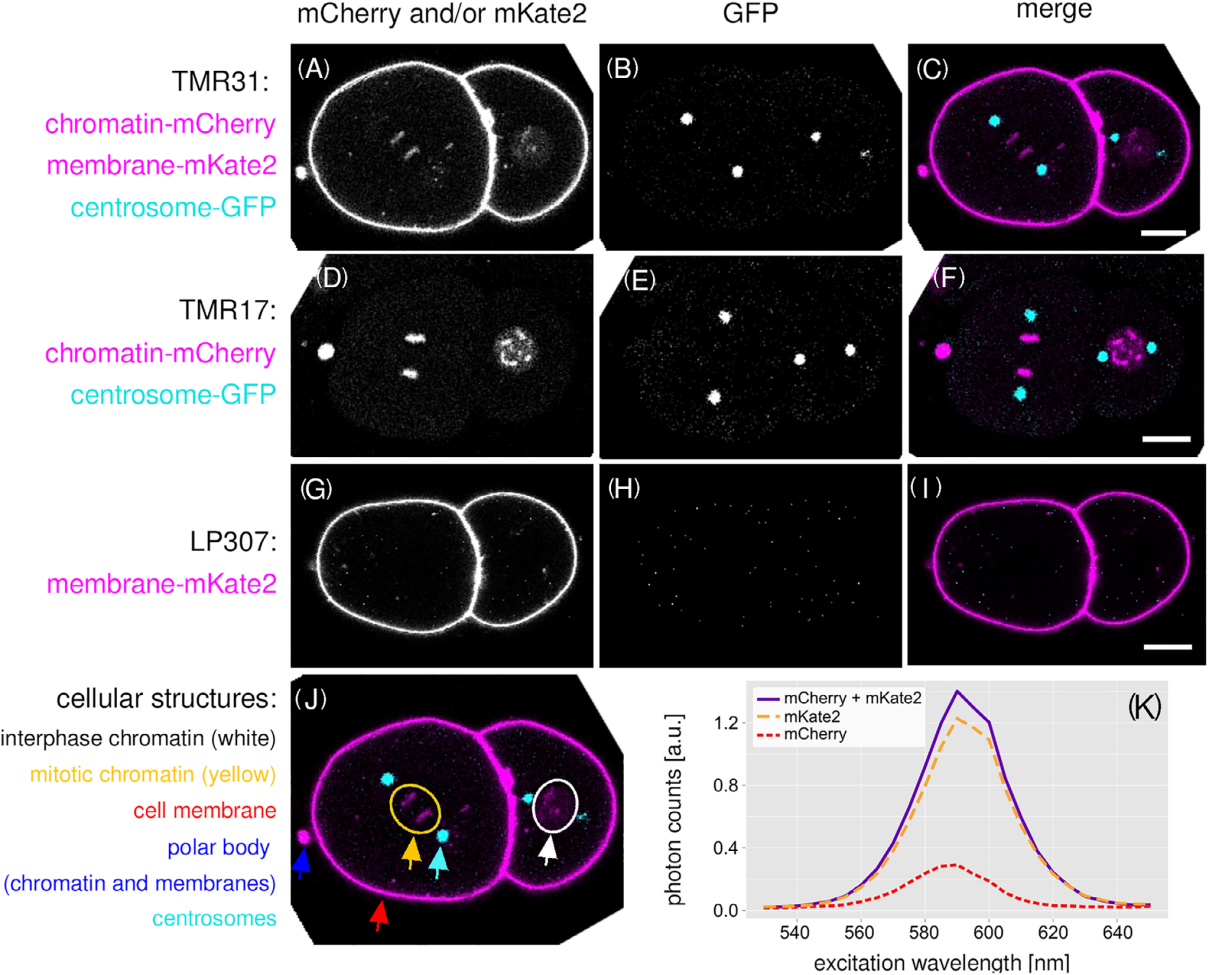
Intensity imaging of worm strains and excitation scans demonstrate the spectral similarity of the red fluorophores mKate2 and mCherry and cellular localisation in embryos. (A–J) Single images of summed intensity stacks of *C. elegans* lines single‐ or double‐positive for chromatin‐mCherry and membrane‐mKate2 show localisation of fluorescence to chromatin and membranes, respectively, in 2‐cell embryos. Centrosome‐GFP further marks the mitotic spindles (columns 2 and 3). (J) Triple fluorescent embryos highlight interphase (white ellipse) and mitotic chromatin (yellow ellipse), cell membranes (red arrow), a polar body (blue arrow) with dense co‐presence of chromatin and membrane and centrosomes (cyan arrow). Scale bars, 10 µm. (K) Excitation scans in the range of 530 to 650 nm in single‐ and double‐positive embryos demonstrate spectral overlap of fluorescence of mCherry and mKate2 (link to Figure 1: https://omero.med.tu‐dresden.de/figure/file/5748/).

**FIGURE 2:**
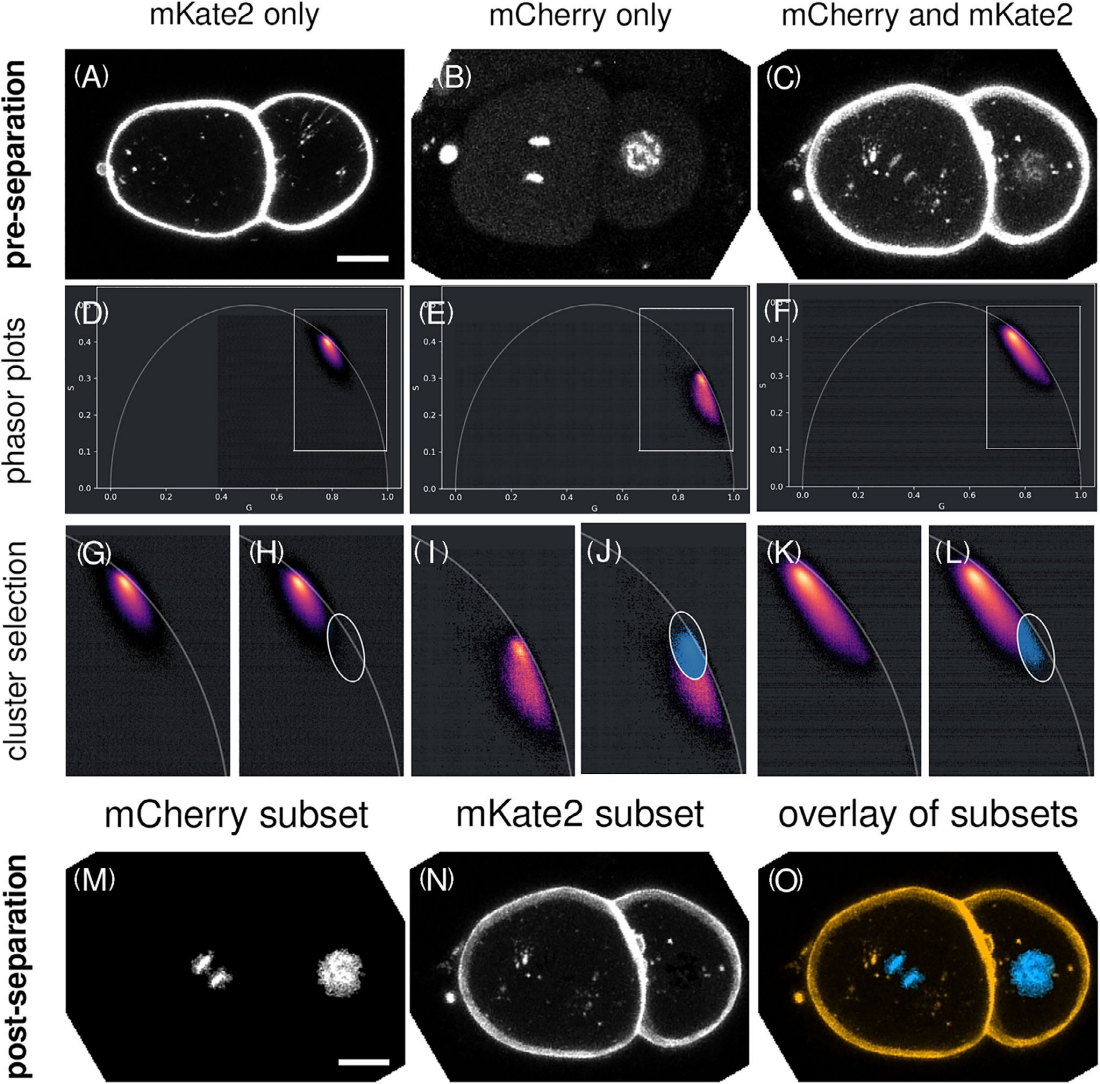
Separation of image stacks into distinct sub‐datasets of spectrally similar fluorophores by lifetime separation. (A, B) Sub‐stack of images of an embryo tagged with mKate2 or mCherry only and (C) an embryo with mCherry and mKate2 signals. (D–F) Corresponding full lifetime phasor plots. (G–L) Regions of interests of the respective phasor plots of (D–F) with ellipse drawn to select a subset of pixels for lifetime separation (H, J, L). The double‐labelled images (C) were further processed. (M) Lifetime‐separated dataset highlighting the mCherry‐tagged chromatin. (N) Lifetime‐separated dataset highlighting the mKate2‐tagged cell membrane. (O) Lifetime‐separated dataset corresponding to (C) with overlaid chromatin (blue) and cell membrane (orange). The separation was based on the selected signals from the phasor plot (L). Scale bar, 10 µm. Sub‐stack planes 19–28, time‐point 6 (A), planes 16–25, time‐point 15 (B) and planes 19–28, time point 10 (C) (link to Figure 2: https://omero.med.tu‐dresden.de/figure/file/5749/).

**FIGURE 3:**
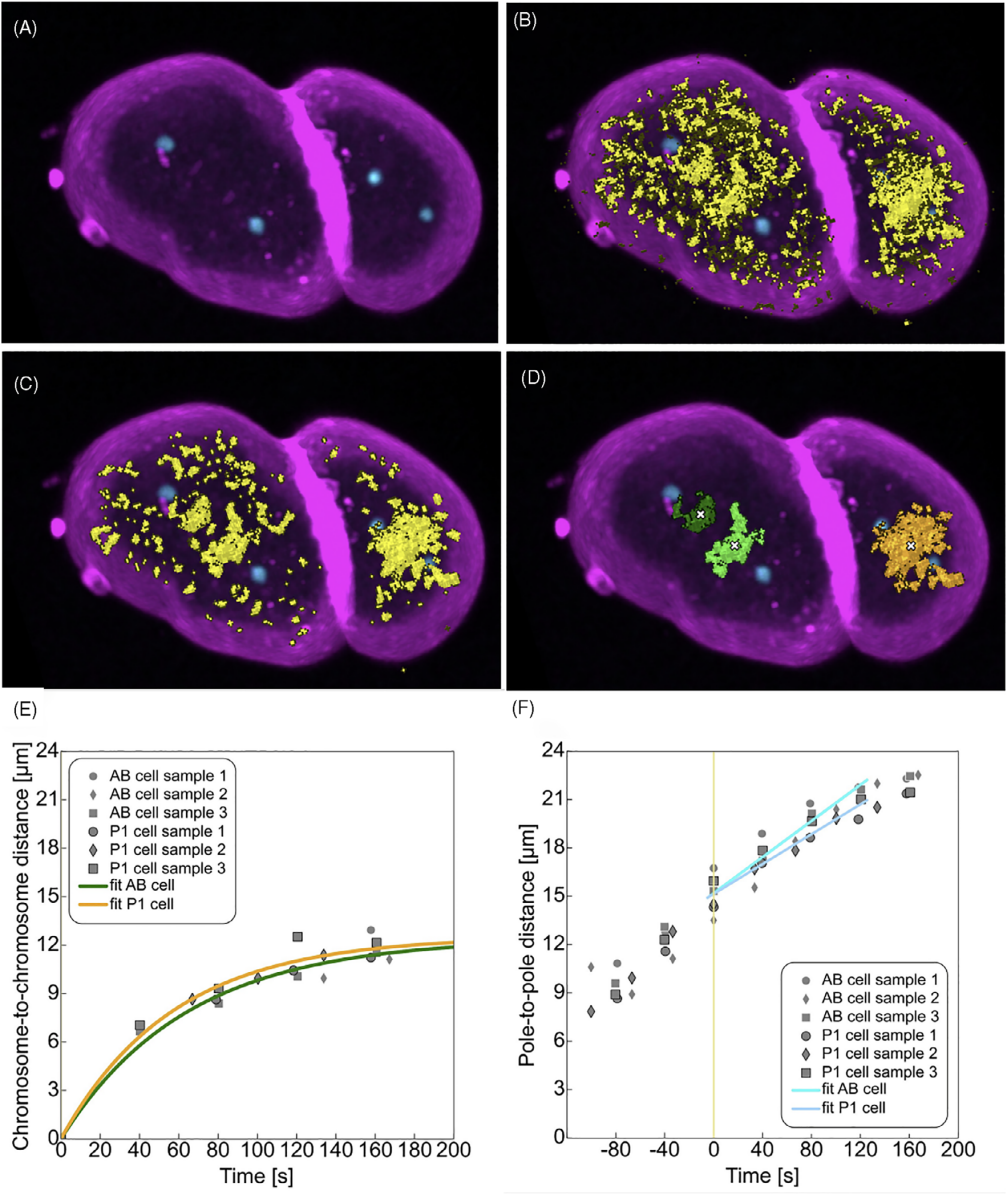
Workflow from lifetime‐separated mCherry channel using the NFPP plugin for analysis of spindle dynamics. (A) Maximum intensity projection of a confocal image stack of one TMR31 *C. elegans* embryo (time point 10) double positive for chromatin‐mCherry and membrane‐mKate2 (both in magenta) combined with centrosome‐GFP (cyan), 3D rendered with napari. (B) Same image as in (A) overlayed by a yellow mask resulting from ellipse cluster selection from the phasor plot. (C) Image with phasor mask post‐processed by the NFPP plugin function ‘smooth cluster mask’: fill holes (scikit‐image ‘area closing’) + connect nearby objects (scikit‐image ‘isotropic closing’) + remove small objects (scikit‐image ‘isotropic opening’). (D) Instance segmentation on post‐processed phasor mask from (C) by connected components labelling (scikit‐image ‘label’) followed by removing objects smaller than volume threshold (pyclesperanto prototype ‘exclude small labels’). The white crosses indicate the centre of the segments used for the determination of the distance of chromosomes over time. For scale, the length of the *C. elegans* embryo along the long embryo axis is about 50 µm. (E) Plot showing chromosome‐to‐chromosome distance over time for the AB‐ and P1‐cell in each analysed TMR31 embryo (grey symbols) along with exponential curves fitted to the measurements (green and yellow). For fitting a function of the form f(*x*) = *a* (1 – *e* – *x*/*b*) was used where *a* = 12.4239 and *b* = 64.0490 for the AB cell and *a* = 12.5410 and *b* = 56.9436 for the P1 cell.  (F) Plot showing pole‐to‐pole distance over time for the AB and P1‐cell in each analysed TMR31 embryo (grey symbols) along with linear curves fitted to the measurements (cyan and light blue) from anaphase onset (*t* = 0) until 120 s after anaphase onset. For fitting a function of the form f(*x*) = *ax*+*b* was used where *a* = 0.0560 and *b* = 15.1896 for the AB cell and *a* = 0.0463 and *b* = 15.1462 for the P1 cell (link to Figure 3: https://omero.med.tu‐dresden.de/figure/file/5819/).

**FIGURE 4:**
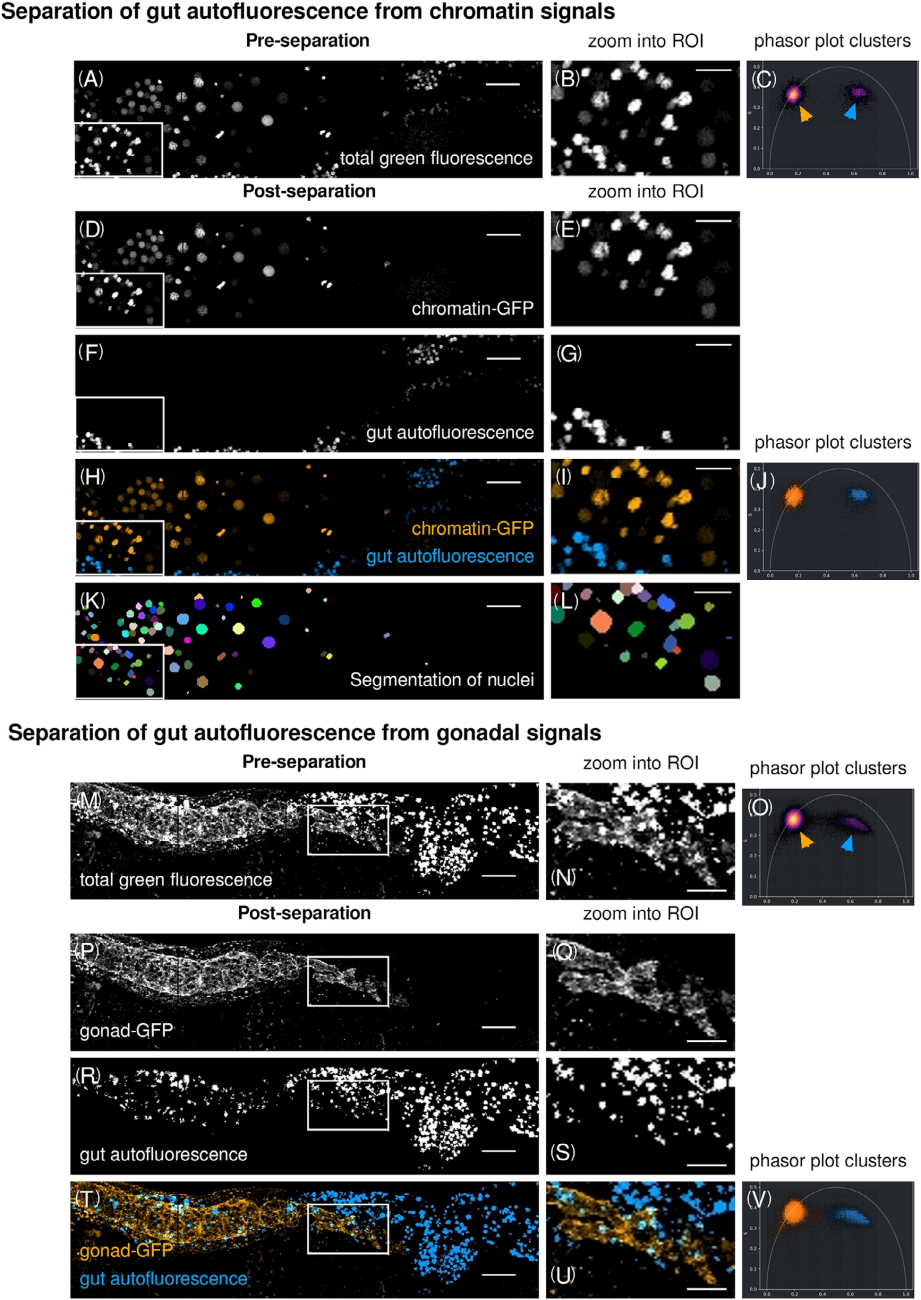
Removal of gut autofluorescence from images of adult *C. elegans* hermaphrodites by lifetime separation. (A, B) Chromatin‐GFP and gut autofluorescence both emit in the GFP spectrum. (C) NFPP‐created phasor plot showing two distinct populations of fluorescence (orange and blue arrow). Lifetime separation creates distinct datasets of GFP‐tagged chromatin (orange) and gut autofluorescence (blue) as a basis for pseudo‐channel‐specific segmentation of cell nuclei. (D, E) Chromatin‐GFP signals separated by lifetime separation. (F, G) Gut autofluorescence separated by lifetime separation. (H, I) Overlay of separated chromatin‐GFP signals (orange) and gut autofluorescence (blue). (J) NFPP‐created phasor plot showing two the two fluorescence populations selected (orange and blue clusters). (K, L) Segmentation of GFP‐positive lifetime‐separated nuclei using napari. (M, N) Gonad‐GFP and gut autofluorescence both emit in the GFP spectrum. (O) NFPP‐created phasor plot showing two distinct populations of fluorescence (orange and blue arrow). Lifetime separation creates distinct datasets of the GFP‐tagged gonad (orange) and gut autofluorescence (blue) as a basis for pseudo‐channel‐specific segmentation of the gonad. (P, Q) Gonad‐GFP signals separated by lifetime separation. (R, S) Gut autofluorescence separated by lifetime separation. (T, U) Overlay of separated gonad‐GFP signals (orange) and gut autofluorescence (blue). (V) NFPP‐created phasor plot showing the two fluorescence populations selected (orange and blue clusters). Scale bars, 20 µm in overview images (first column) and 10 µm in magnified regions of interest (second column) (link to Figure 4: https://omero.med.tu‐dresden.de/figure/file/5820/).

For the generation of figures (Figures 1, 2, 3, 4), we used OMERO.figure (https://www.openmicroscopy.org/omero/figure/). This provides access to the original and processed datasets directly through hyperlinks in the composed figures. For depiction according to conventions, images of embryos were rotated in a stereotypical way with the larger (AB) cell always positioned on the left and the smaller (P1) cell always on the right.

## 3 RESULTS

### 3.1 Characterisation of fluorophores with overlapping spectra

To visualise dynamics of cellular processes during cell divisions, we imaged *C. elegans* embryos simultaneously expressing chromatin‐mCherry, membrane‐mKate2 and centrosome‐GFP. As both fluorophores, mCherry and mKate2 emit in the red light spectrum, there was substantial spectral overlap limiting the number of simultaneously detectable macromolecules. We first characterised the spectral overlap of red fluorophores by image visualisation in conventional confocal microscopy mode as 3D intensity image stacks at a single time point (Figure 1, https://omero.med.tu‐dresden.de/figure/file/5748/). The mCherry‐tagged protein labelled indeed the chromatin of cells in either interphase or mitosis (Figure 1A–F and J), while the mKate2‐tagged protein marked the membrane of the embryonic cells (Figure 1A–C, G–I, and J). Both histone‐mCherry and membrane‐mKate2 fluorescence were detected also at the extruded polar bodies (Figure 1J). In addition, we confirmed the labelling of the spindle poles by centrosome‐GFP (Figure 1A–F and J). Fluorescence imaging further revealed a difference in local intensity of the chromatin‐mCherry versus the membrane‐mKate2 signal. The chromatin signal was much weaker compared to the membrane signal, an observation we could confirm by spectral scans (Figure 1K). Emission scans across a broad excitation range confirmed the spectral similarity of mCherry and mKate2 fluorescence in the imaged worms.

### 3.2 Lifetime separation of spectrally similar fluorophores using the napari‐flim‐phasor‐plotter (NFPP) and napari

To demonstrate the applicability of the NFPP plugin in live‐cell imaging, we performed lifetime separation of mCherry and mKate2‐tagged proteins during mitosis in early *C. elegans* embryos. Using NFPP, we separated the superimposed red fluorescence signals of the chromosomes and of the membranes into fluorophore‐specific pseudo‐channels. Here, NFPP was used for the import, visualisation and processing of multi‐dimensional FLIM datasets of several conventional formats including SDT, PTU and TIFF. The plugin thus extends the N‐dimensional python‐based image viewer napari, a powerful community‐driven open‐source platform for image visualisation and analysis, to FLIM datasets.

To separate FLIM datasets into distinct pseudo‐channels, we generated 6D FLIM datasets of xyzctµ dimensions, being x, y and z spatial dimensions, as well as dual channels (c), a time‐series (t) and FLIM microtime (µ) dimension (Figure 2, https://omero.med.tu‐dresden.de/figure/file/5749/). We show intensity images of maximum projections of single time‐point sub‐stacks of early developing *C. elegans* embryos carrying single or double red fluorophores (Figure 2A–C).

The exported data of the acquisition system was then imported into napari using NFPP making use of python libraries for native FLIM file import. The plugin further converted the lifetime information per voxel into a 2D plot termed phasor plot by Fourier transformation (Figure 2D–F). Magnifications of regions of interest of phasor plots emphasise the difference in lifetime‐based localisation of fluorophore‐specific data points visualised as density plots (Figure 2G, I, and K) that are a prerequisite for successful lifetime separation of datasets by selection of phasor clusters (Figure 2H, J, and L). Due to the high abundance of mKate2‐positive voxels, the signal of mCherry‐positive data points is barely visible in the phasor plots (Figure 2F, K, and L) which emphasises the importance of single fluorophore controls (Figure 2D, G, and H and Figure 2E, I, and J). Overall, red fluorescence of distinct fluorophores could be separated into sub‐datasets of chromatin and membrane signal by lifetime separation using NFPP (Figure 2M–O).

### 3.3 Lifetime separation for live imaging of cell division

Next, we investigated the dynamics of chromosomes and centrosomes during mitosis in two‐cell *C. elegans* embryos by downstream analysis of lifetime‐separated datasets in napari (Figure 3, https://omero.med.tu‐dresden.de/figure/file/5819/). For that, the aforementioned method was used to process the raw data (Figure 3A) and segment the mCherry‐positive voxels in the phasor plot (Figure 3B). Data was then filtered, resulting in much less segmented voxels (Figure 3C) which enabled a selection of those segments containing the chromosomes (Figure 3D). We then calculated the centres of mass of each segment (Figure 3D, white crosses) and used these coordinates to determine the chromosome‐to‐chromosome distance over time (Figure 3E). By fitting an exponential curve we determined a speed of 11.64 µm/min for the chromosomes in the larger AB cell and a speed of 13.21 µm/min in the smaller P1 cell of the two‐cell stage embryo. For centrosomes, we used adaptive local thresholding for segmentation and used the resulting centre coordinates to calculate the pole‐to‐pole distances over time (Figure 3F). By fitting a linear function to the pole‐to‐pole distance after anaphase onset until 120 s after anaphase onset, we determined a speed of pole separation of 3.36 µm/min for the AB cell and 2.78 µm/min for the P1 cell. These values matched previously published data on centrosome separation in the two‐cell embryo.^63^ Hence, we demonstrated that lifetime‐separated live‐cell bioimages can be applied to precisely track dynamic processes during animal development.

### 3.4 Removal of autofluorescence from bioimage datasets by lifetime separation

We further evaluated the applicability of the established workflow of lifetime separation using the NFPP plugin to split natural autofluorescence from spectrally overlapping GFP fluorescence marking biomolecules of interest in adult worms (Figure 4, https://omero.med.tu‐dresden.de/figure/file/5820/). Strong intrinsic autofluorescence of biological samples is often a challenge in imaging of both tissues and whole organisms and can mask and hinder the analysis of cellular processes of interest. Biomolecules such as chitin, melanin, NADH and FAD and many others are fluorescent in green or red spectral ranges.^4^, ^64^, ^65^, ^66^ Such biomolecules with structural, protective and/or metabolic functions can occur at high concentrations across tissues and also whole organisms.^6^ As an example, the gut of worms is highly autofluorescent, thus hindering reliable microscopic analysis of nearby tissues such as the gonad.

We imaged adult hermaphrodites with either chromosome‐GFP (Figure 4A–J) or gonad‐GFP (Figure 4K–R) fluorescent proteins, both known to be in close proximity to the gut as a source of animal‐intrinsic autofluorescence. We could clearly distinguish nuclei of the gonad in the chromosome‐GFP worm line. Lifetime separation enabled removal of the gut autofluorescence and subsequent segmentation of the gonadal nuclei for potential further analysis (Figure 4C, D, and I–L). In addition, removal of gut autofluorescence from worms expressing gonad‐GFP (Figure 4M and N) allowed the distinct localisation and segmentation of the membrane and other features of the gonad (Figure 4M–R). Compared to conventional intensity imaging, removal of autofluorescence by lifetime separation improves the localisation of biomolecules of interest and thus the quality of scientific output from live‐cell imaging across scales.

## 4 DISCUSSION

FLIM contributes a valuable additional dimension of information to bioimaging beneficial for the identification, characterisation and/or separation of fluorophores in their immediate environment. Here, we present lifetime separation for the distinction of sample‐intrinsic and ‐extrinsic fluorophores in developing live organisms using a newly developed napari plugin, NFPP. This plugin feeds datasets with a lifetime decay dimension into napari, linked to first modes of lifetime analysis and conversion of FLIM datasets to ZARR format for downstream analysis and visualisation. Our work emphasises the current challenges and first solutions for FLIM in general and in 6D datasets in particular. This imaging technique demands a carefully designed acquisition setup combined with enhanced interoperability of data formats and analysis workflows to simplify and standardise its application.

### 4.1 Acquisition of FLIM data

**FIGURE 5:**
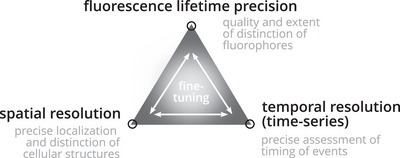
The triangle of compromise in FLIM imaging. Levels of resolution in lifetime, space and time‐series resolution require careful and project‐specific fine‐tuning to obtain the required level of information across all dimensions.

FLIM requires a careful balance of lifetime, spatial and temporal resolution (Figure 5). In detail, the detection of sufficient photons is required for precise lifetime decay estimations. Despite the constantly increasing sensitivity of detectors, FLIM requires relatively long image acquisition times compared to corresponding intensity measurements. This challenges particularly multi‐dimensional imaging of dynamic processes in living cells as temporal resolution, both in lifetime and time series dimension, and spatial resolution have to be levelled carefully. For example, higher photon counts in the lifetime dimension render decay curves less noisy, leading to more compact and thus distinct phasor clusters and more precise lifetime calculations compared to lower photon count scenarios. Specifically, if the lifetimes of fluorophores are similar, showing differences of less than one nanosecond, separation may require a high number of photons. In addition, a phasor cluster of autofluorophores with large variation due to structural variability of the fluorochrome or diverse interaction partners may thus require a high number of photons.

In general, the size of investigated structures defines the required resolution in x, y and z dimensions. Pixel binning might be an option to improve the lifetime precision, if the investigated structures can still be identified. Furthermore, the temporal resolution of a dynamic process is also a critical parameter. As for the investigation of chromatin dynamics in our study, the distinction of individual chromosomes was essential to track their dynamics during separation. However, the density of chromosomal proteins in interphase versus mitosis clearly differs, consequently leading to different photon counts across pixels and thus different quality of phasor clusters and pixel classification. In addition, investigation of overall less abundant proteins with spectrally similar tags might further challenge the pixel classification based on phasor clusters. It is, therefore, important to fine‐tune the individual steps of the workflow, including data acquisition, lifetime separation and image analysis. Only then, FLIM is a powerful imaging technique for the multi‐dimensional investigation of dynamic processes.

### 4.2 Handling and storage of FLIM data

**FIGURE 6:**
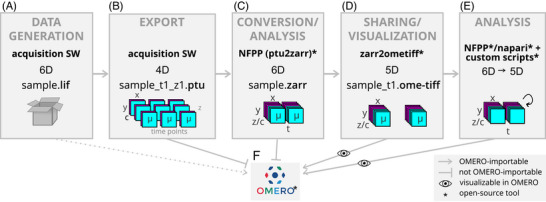
Workflow of FLIM file format conversion for sharing and analysis. (A) Storage of 6D FLIM datasets in LIF format using proprietary acquisition software. (B) Export of data in 4D sub‐datasets in PTU format split by z planes and time points. (C) Reassembly of datasets to 6D using the napari‐flim‐phasor‐plotter (NFPP) plugin, and optionally conversion to ZARR. (D) Conversion to the OME‐TIFF format for 5D datasets split by time points permits OMERO upload of image data and metadata. (E) Reduction of 6D data in ZARR format to 5D by FLIM analysis using NFPP along with napari and custom scripts contributes additional segmentation masks to 5D datasets in OMERO. (F) Overview of possible import of data including segmentation results based on lifetime separation into OMERO and possibility of visualisation of lifetimes as time series. Lines with arrows indicate the option for OMERO upload of each dataset in its format and number of dimensions. Blocked lines indicate no current option for OMERO upload limited by format (PTU) or 6 dimensions (ZARR). Files in LIF format can be formally uploaded but do not display properly. Eye symbols indicate proper visualisation of datasets in OMERO. Asterisks indicate open‐source tools and scripts. x and x and y spatial dimensions; z, z spatial dimension; c, channel; t, time dimension; µ, photon counts in lifetime dimension (microtime); SW, software; NFPP, napari‐flim‐phasor‐plotter plugin.

Critical for a broad range of FLIM applications are aspects concerning data handling and analysis as exemplified here for 6D datasets (Figure 6). First, FLIM data is to a large extent stored in less abundant and thus less supported formats including PTU and SDT. This hinders implementation of proper handling of FLIM data along the data life cycle ranging from import to visualisation, analysis, sharing for publication and reuse and archiving. Processing of lifetime information demands specialised workflows, most prominently exponential fitting or phasor analysis based on Fourier transformation of decay information, essential for correlation with further measurements. Thus, FLIM analysis requires extensive integration into image analysis tools with various functionalities to adjust to research questions.

As previously mentioned, there are a couple open‐source options available for FLIM analysis using the fitting approach (FLIMfit^18^ and FlimJ^19^), while there has been a clear recent engagement into the phasor approach, probably due to its user‐friendlier usage. PAM,^20^ a software written in MATLAB, has a lot of methods for FLIM analysis and was probably the first to bring phasor analysis as one of them. Just a couple years ago, FLUTE^21^ was published as a stand‐alone open‐source software specifically for phasor analysis. It provides a user‐friendly interface and offers tools to visualise and filter regions with different lifetimes by means of cursors and other filtering tools. Around the same time, PhasorIdentifier^22^ was published as a cloud‐based solution for phasor analysis via tailored Jupyter notebooks that could be run via Google Colab. The interface involves running these notebooks to display a few widgets after setting the input variables in the beginning. Napari‐live‐flim^23^ came out as the first publication of a napari plugin for FLIM analysis. It was designed to conduct FLIM analysis in real‐time via temporary files sent from Micro‐Manager.^67^ Napari‐phasors^25^ uses the phasorpy library^24^ to read FLIM data, calculate phasors to display them inside napari for manual selection. More recently, GSLab^26^ was published as a platform for phasor analysis written in MATLAB, made available via a compiled Windows installer. It provides phasor separation using higher harmonics and automated phasor clustering with Gaussian Mixed Models. Finally, FLIMPA^27^ is to date the most recent stand‐alone software. It can, among other things, run FLIM TCSPC analysis from a couple proprietary file formats and plot phasors for multiple samples. Each of these tools has potentially unique features that should be considered depending on the desired analysis. Our plugin offers unique support for processing high‐dimensional FLIM data, allowing researchers working with time‐lapse 3D stacks to segment samples with different lifetimes. It also provides the option to convert to file formats compatible with open image data repositories like OMERO.

Thus, in our work, we present a comprehensive approach for 6D FLIM data analysis with increased implementation of FAIR principles to make FLIM datasets more findable, accessible, interoperable and reusable.^68^ In our study, datasets were initially stored in LIF format with limited lifetime visualisation in OMERO (Figures 6A and S1) and acquisition software‐restricted export of only split datasets in PTU format separated in z planes and time points (Figure 6B) with partially lost corresponding metadata. Split datasets were reassembled and reshaped into 6D during import into napari using NFPP (Figure 6C).

To date, sharing of FLIM data of various formats via OMERO is still challenged by the restricted availability of readers for specialised and less abundant formats and the limitation of dimensionality (Figure 6F). Thus, while datasets in PTU format are not importable to OMERO (Figure 6C and F), those of LIF format lack visualisation of detailed lifetime decays but offer instead processed lifetime information and a Fast FLIM view (Figure 6A and F). Datasets of ZARR and OME‐TIFF formats are importable in 5D (Figures 6C, D and F and S2) with improved metadata annotation in OME‐TIFF format. FLIM‐based segmentation masks can enrich corresponding datasets in OMERO (Figures 6E and S2). The community‐driven development of next‐generation bioimage file formats (https://ome‐model.readthedocs.io/en/stable/developers/6d‐7d‐and‐8d‐storage.html) will likely simplify the handling of FLIM datasets along the data life cycle including visualisation, integrative lifetime analysis and sharing according to the FAIR principles in the future.

In summary, this study demonstrates the successful application of FLIM for lifetime separation of multiplexing fluorophores applied for live‐cell imaging of dynamic processes in both *C. elegans* embryos and adult animals. Combining improved and transparent data handling and analysis workflows, this interdisciplinary project aims to broaden the application and the scientific readout of FLIM imaging in the life sciences.

## Supporting information




**FIGURE S1** Images of FLIM datasets in LIF format in OMERO. (A) List of images as part of each imported 6D LIF dataset as visualised in OMERO. (B) Images show for example phasor parameters or FAST FLIM lifetimes, a single FLIM‐based value per voxel, but no lifetime information as separate dimension. Look‐up table settings are chosen for best visual contrast of the respective parameters for the datasets.

**FIGURE S2** Images of 5D FLIM datasets in OME‐TIFF format in OMERO. (A) Thumbnails of a 5D intensity image dataset with lifetime‐based segmentation mask of chromatin as well as a 5D dataset with a lifetime slider, separated by time points (combined mCherry and mKate2 in magenta and centrosomes in cyan). (B–D) Detailed views of a single plane showing an exemplary 5D intensity dataset with NFPP‐based segmentation of chromatin (B) and an exemplary single time point single plane of a 5D FLIM dataset (C) at different exemplary time points during exponential lifetime decay time points visualised in an OMERO.figure panel (D).
