# Supplementary material for: A fluorescence lifetime separation approach for FLIM live‐cell imaging
Source: J Microsc. 2025 Sep 30;301(1):91–106. doi: 10.1111/jmi.70036 (PMC12746358; doi:10.1111/jmi.70036)
Supplement: Supplementary file 1 — FIGURE S1 Images of FLIM datasets in LIF format in OMERO. (A) List of images as part of each imported 6D LIF dataset as visualised in OMERO. (B) Images show for example phasor parameters or FAST FLIM lifetimes, a single FLIM‐based value per voxel, but no lifetime information as separate dimension. Look‐up table settings are chosen for best visual contrast of the respective parameters for the datasets. [file JMI-301-91-s001.pdf]

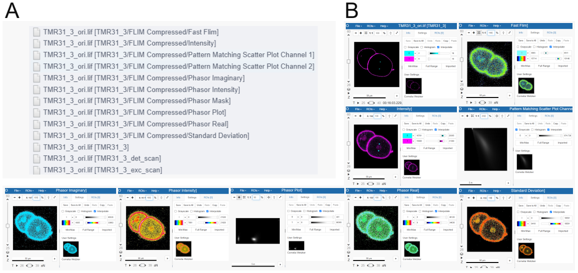
A
TMR31_3_ori.lif [TMR31_3/FLIM Compressed/Fast Flim]
TMR31_3_ori.lif [TMR31_3/FLIM Compressed/Intensity]
TMR31_3_ori.lif [TMR31_3/FLIM Compressed/Pattern Matching Scatter Plot Channel 1]
TMR31_3_ori.lif [TMR31_3/FLIM Compressed/Pattern Matching Scatter Plot Channel 2]
TMR31_3_ori.lif [TMR31_3/FLIM Compressed/Phasor Imaginary]
TMR31_3_ori.lif [TMR31_3/FLIM Compressed/Phasor Intensity]
TMR31_3_ori.lif [TMR31_3/FLIM Compressed/Phasor Mask]
TMR31_3_ori.lif [TMR31_3/FLIM Compressed/Phasor Plot]
TMR31_3_ori.lif [TMR31_3/FLIM Compressed/Phasor Real]
TMR31_3_ori.lif [TMR31_3/FLIM Compressed/Standard Deviation]
TMR31_3_ori.lif [TMR31_3]
TMR31_3_ori.lif [TMR31_3_det_scan]
TMR31_3_ori.lif [TMR31_3_exc_scan]
B
TMR31_3_ori.lif [TMR31_3]
Fast Flim]
Intensity]
Pattern Matching Scatter Plot Channel
Phasor Imaginary]
Phasor Intensity]
Phasor Plot]
Phasor Real]
Standard Deviation]
