# Supplementary figures and images for: A fluorescence lifetime separation approach for FLIM live‐cell imaging
Source: J Microsc. 2025 Sep 30;301(1):91–106. doi: 10.1111/jmi.70036 (PMC12746358; doi:10.1111/jmi.70036)

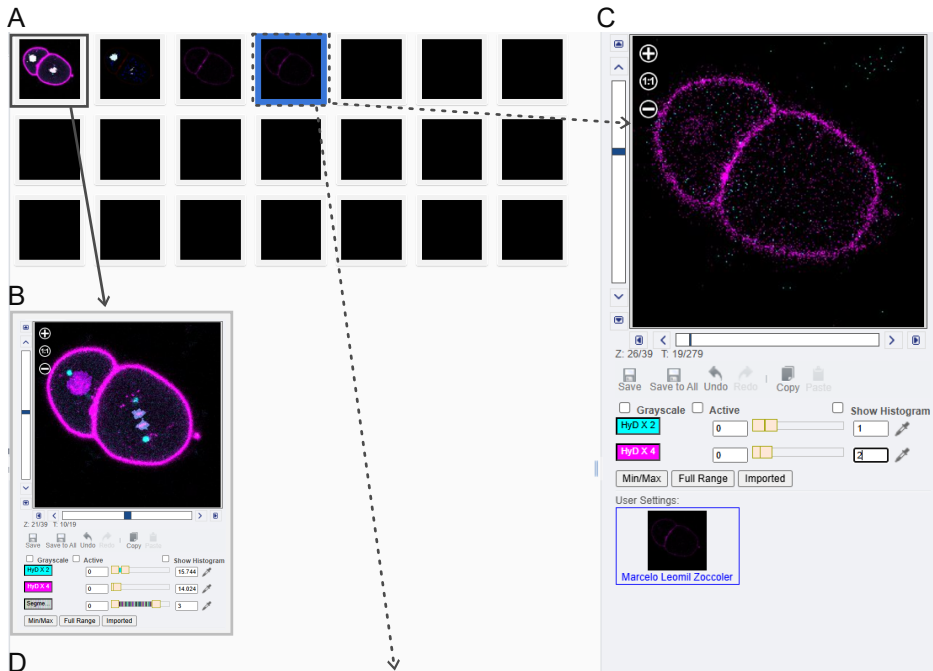

Supplement: Supplementary file 2 — FIGURE S2 Images of 5D FLIM datasets in OME‐TIFF format in OMERO. (A) Thumbnails of a 5D intensity image dataset with lifetime‐based segmentation mask of chromatin as well as a 5D dataset with a lifetime slider, separated by time points (combined mCherry and mKate2 in magenta and centrosomes in cyan). (B–D) Detailed views of a single plane showing an exemplary 5D intensity dataset with NFPP‐based segmentation of chromatin (B) and an exemplary single time point single plane of a 5D FLIM dataset (C) at different exemplary time points during exponential lifetime decay time points visualised in an OMERO.figure panel (D). [file JMI-301-91-s002.pdf]
